# Room-temperature macromolecular serial crystallography using synchrotron radiation

**DOI:** 10.1107/S2052252514010070

**Published:** 2014-05-30

**Authors:** Francesco Stellato, Dominik Oberthür, Mengning Liang, Richard Bean, Cornelius Gati, Oleksandr Yefanov, Anton Barty, Anja Burkhardt, Pontus Fischer, Lorenzo Galli, Richard A. Kirian, Jan Meyer, Saravanan Panneerselvam, Chun Hong Yoon, Fedor Chervinskii, Emily Speller, Thomas A. White, Christian Betzel, Alke Meents, Henry N. Chapman

**Affiliations:** a Center for Free Electron Laser Science, DESY, Notkestrasse 85, Hamburg 22607, Germany; b Institute of Biochemistry and Molecular Biology, University of Hamburg, Hamburg 22607, Germany; c Photon Science, DESY, Hamburg 22607, Germany; d Department of Physics, University of Hamburg, Luruper Chaussee 149, Hamburg 22607, Germany; e European XFEL GmbH, Albert Einstein Ring 19, Hamburg 22761, Germany; f Moscow Institute of Physics and Technology, 141700 Moscow, Russian Federation; g Department of Physics, University of York, Heslington, York YO10 5DD, UK; h Center for Ultrafast Imaging, Luruper Chaussee 149, Hamburg 22761, Germany

**Keywords:** serial crystallography, room-temperature protein crystallography, radiation damage, *CrystFEL*, microfocus beamline

## Abstract

A new approach for collecting data from many hundreds of thousands of microcrystals using X-ray pulses from a free-electron laser has recently been developed. Referred to as serial crystallography, diffraction patterns are recorded at a constant rate as a suspension of protein crystals flows across the path of an X-ray beam. Events that by chance contain single-crystal diffraction patterns are retained, then indexed and merged to form a three-dimensional set of reflection intensities for structure determination. This approach relies upon several innovations: an intense X-ray beam; a fast detector system; a means to rapidly flow a suspension of crystals across the X-ray beam; and the computational infrastructure to process the large volume of data. Originally conceived for radiation-damage-free measurements with ultrafast X-ray pulses, the same methods can be employed with synchrotron radiation. As in powder diffraction, the averaging of thousands of observations per Bragg peak may improve the ratio of signal to noise of low-dose exposures. Here, it is shown that this paradigm can be implemented for room-temperature data collection using synchrotron radiation and exposure times of less than 3 ms. Using lysozyme microcrystals as a model system, over 40 000 single-crystal diffraction patterns were obtained and merged to produce a structural model that could be refined to 2.1 Å resolution. The resulting electron density is in excellent agreement with that obtained using standard X-ray data collection techniques. With further improvements the method is well suited for even shorter exposures at future and upgraded synchrotron radiation facilities that may deliver beams with 1000 times higher brightness than they currently produce.

## Abstract 2

The room-temperature structure of lysozyme is determined using 40000 individual diffraction patterns from micro-crystals flowing in liquid suspension across a synchrotron microfocus beamline.

## 1. Introduction   

X-ray crystallography is the method of choice for protein structure determination. Almost 90% of the more than 95 000 structures in the Protein Data Bank (http://www.rcsb.org; Berman *et al.*, 2000 ▶) have been solved by X-ray methods. There are, however, various bottlenecks to the standard crystallographic pipeline. One of the most serious is the difficulty of growing suitably large and well diffracting crystals, which are needed in order to acquire a sufficient diffraction signal within an X-ray exposure that is limited by the onset of structural disorder caused by that exposure.

Crystal size and radiation damage are inherently linked. Damage due to ionizing radiation depends on dose (that is, energy absorbed per unit mass), which in the case of kinematic diffraction is directly proportional to the incident fluence (photons per unit area). The diffraction signal for a given incident X-ray fluence scales with the illuminated volume of the well ordered crystal, so smaller crystals require higher dose to achieve similar diffraction signals. The tolerable dose of a typical protein crystal at cryogenic temperatures is about 10 MGy per ångstrom of resolution (*i.e.* 30 MGy for the typical 3 Å resolution of protein structures) (Howells *et al.*, 2009 ▶; Holton, 2009 ▶; Owen *et al.*, 2006 ▶). At this dose the structural disorder initiated by photoabsorption causes the highest resolution diffraction intensities to be reduced by half. The tolerable dose for room-temperature measurements is about 30 times less. However, the damage processes at room temperature are dictated by complex processes of radiolysis in liquids and may be dependent on dose rate (Blake *et al.*, 1962 ▶; Southworth-Davies *et al.*, 2007 ▶; Owen *et al.*, 2012 ▶; Warkentin *et al.*, 2011 ▶, 2013 ▶).

When large crystals are not available, a common strategy to work within dose limits is to collect data from many small crystals and then to scale and merge the data into one set of structure factors. Microcrystallography is carried out at several beamlines at synchrotron radiation facilities that deliver monochromatic beams of about 10 µm diameter and typically with 10^12^ photons s^−1^ (Smith *et al.*, 2012 ▶). Doses of 10 MGy can be reached in exposures of seconds at such beamlines. A typical experiment at microcrystallography beamlines consists of mounting a sample loop with one or more crystals that are usually no smaller in volume than about 1000 µm^3^. The crystals are then located and centered in the beam in order to collect a partial dataset while carefully monitoring the dose. The process may be repeated on a number of sample loops to obtain enough data to form a complete dataset. Data collection schemes have been developed employing multiple positions on a single crystal (Riekel *et al.*, 2005 ▶), to distribute the delivered energy over a greater volume of the crystal, or for the collection of partial datasets from multiple crystals (Brodersen *et al.*, 2003 ▶). This approach has yielded structures for microcrystals with volumes as small as about 100 µm^3^ (Coulibaly *et al.*, 2007 ▶) and allowed the measurement of diffraction from membrane protein microcrystals without removing them from crystallization plates (Axford *et al.*, 2012 ▶).

In recent years new sample mounting systems for microfocus beamlines have been developed to measure protein crystals *in situ* at room temperature (Axford *et al.*, 2012 ▶; Pinker *et al.*, 2013 ▶; Guha *et al.*, 2012 ▶; Wang *et al.*, 2012 ▶). Specifically, Guha *et al.* built an X-ray compatible multilayer microfluidic protein crystallization platform and used it to crystallize three model proteins and to collect high-resolution diffraction patterns from them, and Pinker *et al.* developed and tested a microfluidic chip for counter-diffusion crystallization and X-ray analysis. These methods simplify the measurement of a small number of diffraction patterns from many individual microcrystals.

The development of X-ray free-electron lasers (FELs) that produce intense pulses of tens of femtoseconds in duration enables data collection from a room-temperature crystal before the rapid onset of disorder (Barty *et al.*, 2012 ▶), at doses far exceeding tolerable doses with synchrotron radiation. This method of serial femtosecond crystallography (SFX) (Chapman *et al.*, 2011 ▶; Boutet *et al.*, 2012 ▶) introduced a number of innovations, such as data collection from a continuously flowing suspension of nanocrystals in a liquid jet (DePonte *et al.*, 2008 ▶; Shapiro *et al.*, 2008 ▶) with high-frame-rate detectors (Philipp *et al.*, 2011 ▶), and software to process millions of detector frames (Barty *et al.*, 2013 ▶) and to merge data from the hundreds of thousands of flagged ‘still snapshot’ single-crystal diffraction patterns (White *et al.*, 2012 ▶, 2013 ▶). The method was first demonstrated on the membrane protein complex photosystem I, at a resolution limited by the long wavelength of 6 Å that was initially available at the Linac Coherent Light Source (LCLS) (Chapman *et al.*, 2011 ▶), and was soon validated at high resolution using lysozyme microcrystals (Boutet *et al.*, 2012 ▶). SFX has since been applied to solving new structures such as *Trypanosoma brucei* cathepsin B at 2.1 Å resolution from *in vivo* grown microcrystals (Koopmann *et al.*, 2011 ▶; Redecke *et al.*, 2013 ▶) and the serotonin receptor 5HT2B from a suspension of microcrystals in a lipidic cubic phase matrix (Liu *et al.*, 2013 ▶). The general paradigm of collecting diffraction patterns from a large number of previously unexposed and uncharacterized crystals was very recently applied at a synchrotron beamline (Gati *et al.*, 2014 ▶) where almost 29 000 diffraction frames were collected in rastered helical scans across a cryogenically cooled suspension of *in vivo* grown crystals of *Trypanosoma brucei* cathepsin B. Each crystal was only 9 µm^3^ in volume. The *CrystFEL* software (White *et al.*, 2012 ▶, 2013 ▶) was used to identify and index single-crystal diffraction patterns in the data stream. Strong patterns collected consecutively on the same crystal were grouped and treated as regular rotation data, enabling structure determination to 3.3 Å resolution, which compares with the 2.1 Å resolution achieved at the LCLS.

Here, we demonstrate that the paradigm of serial crystallography can be successfully applied at a synchrotron beamline by collecting a large number of short exposures from room-temperature microcrystals suspended in their growth medium. In our adaptation, millions of detector frames are acquired at a constant rate while the suspension of crystals continuously flows across the beam in a thin-walled capillary. The beam is not shuttered between exposures, and the actual exposure time of a crystal (and hence the dose it receives) is therefore set by the time it takes for that crystal to transit the X-ray focus. The detector frame rate, sample flow rate and crystal concentration are adjusted to ensure that it is more likely to record diffraction from single crystals rather than multiple crystals in a detector frame. The detector frames are processed using a similar pipeline to that used for SFX, whereby frames are first searched for the presence of crystal diffraction, then indexed and merged into a set of structure factors. This set of structure factors is obtained from a Monte Carlo integration of indexed spots that averages over variations in crystal size, shape, quality, orientation and other variables.

Unlike the femtosecond snapshots of X-ray FEL diffraction, exposures at a synchrotron are for sufficient duration that crystals rotate slightly during exposure (depending on the viscosity of the medium carrying them). This rotation allows for some Bragg peaks to be completely recorded in an individual exposure.

One way to visualize serial crystallography is as powder diffraction, recorded one grain at a time. Collecting individual crystals snapshots allows the orientation of each grain to be determined and background-subtracted from each frame before summation. As with powder diffraction, the resolution limit is where the diffraction signal summed from many individual crystals can no longer be observed above the noise in the background. However, unlike powder diffraction, data frames are collected from individual crystals. Compared with the case of exposing the entire sample in a single powder pattern, this enables the contribution from background to be reduced by rejecting detector frames that do not contain crystal diffraction. In those frames that are selected, the background is proportional to the thickness of the crystal-containing medium and capillary walls as well as the exposure time. Hence, this method can be improved by supplying the suspension in a thin sheet or column, and by setting the detector recording time to be no longer than the crystal transit time.

The outline of this paper is as follows: in §2 we describe our experimental demonstration of serial crystallography at the P11 beamline of the PETRA III facility at DESY, Hamburg, on lysozyme crystals of about 5 µm diameter, measured at a dose of up to 0.3 MGy with crystal transit times of a few milliseconds. The structure at 2.1 Å resolution was obtained from 40 233 indexed patterns selected from over a million detector frames, as described in §3. We examine the dependence of the data quality, structure refinement statistics and resolution on the number of indexed patterns. Our experiments also highlight improvements and further developments to be made, which are discussed in §4. These experiments are a proof-of-principle demonstration of a method that is scalable to much faster data collection using upgraded high-brightness synchrotron sources and detectors that are underway or being planned at many facilities. For example, intense beams of 1.5% bandwidth (‘pink beam’) may soon become available at PETRA III and ESRF that are several orders of magnitude higher intensity than demonstrated here. At such beamlines it may be possible to rapidly collect data with crystal transit times of tens of microseconds, using liquid jets of appropriate speed (DePonte *et al.*, 2008 ▶; Weierstall *et al.*, 2014 ▶) and high-frame-rate detectors (Henrich *et al.*, 2011 ▶; Becker *et al.*, 2013 ▶). The continuous sample delivery inherent to the method is well suited for time-resolved studies of irreversible reaction on timescales matching the crystal transit time, providing a new scheme for measuring dynamics and kinetics of macromolecular crystal structures.

## 2. Materials and methods   

**Figure 1:**
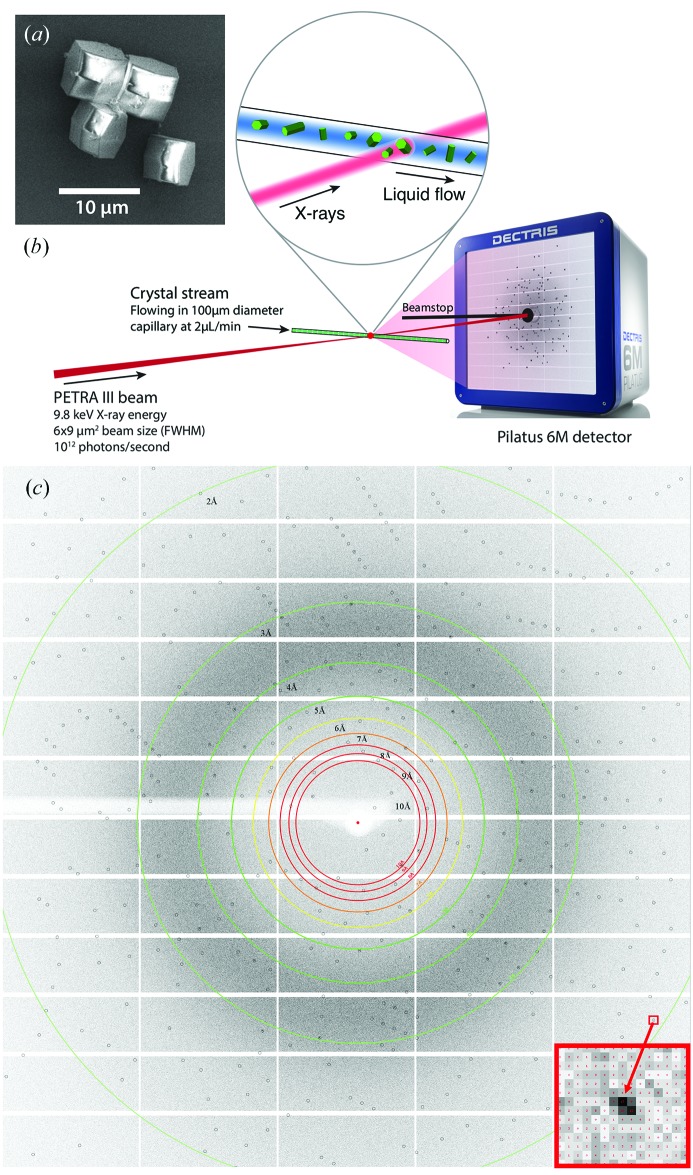
(*a*) SEM micrograph of the lysozyme microcrystals used for the serial crystallography measurements. (*b*) Sketch of the experimental set-up for protein serial crystallography at the PETRA III P11 beamline. (*c*) A single-crystal diffraction pattern. The circles show predicted positions of Bragg peaks. Bragg spots can be observed up to the centre edge of the detector, as shown by the red arrow that indicates a Bragg spot located at 2.05 Å resolution. Photograph of the Pilatus 6M detector by courtesy of DECTRIS Ltd.

We prepared chicken egg-white lysozyme microcrystals in batch mode using a modified version of the protocol described by Falkner *et al.* (2005 ▶), omitting the final cross-linking step. Microcrystals were obtained at room temperature by adding three parts of precipitant [14.7%(*w*/*v*) NaCl, 22%(*w*/*v*) PEG 8000, both from Sigma Aldrich, Germany, in 500 m*M* acetate buffer at pH 3] to one part of lysozyme (Sigma Aldrich, Germany, 100 mg ml^−1^ in the same buffer) followed by immediate thorough stirring for two minutes. All solutions were prepared using ultrapure water and filtered through a 0.1 µm filter (Sartorius Stedim, Germany) prior to crystallization. A high-concentration suspension of about 5 × 10^7^ microcrystals ml^−1^ was obtained after incubation for 12 h. Absorption photometry at 280 nm wavelength using a NanoDrop 2000c (Peqlab, USA) revealed that less than 0.05 mg ml^−1^ lysozyme remained in solution after crystallization. Prior to data collection the crystal concentration was increased by a factor of two by centrifugation and the PEG concentration of the suspension was adjusted to 28%(*w*/*v*) to prevent settling in the sample reservoir and in the capillary during the measurements. We observed that the crystals were prisms with an edge length of about 3 µm and a long axis of about 6 µm, thus with an average volume of about 135 µm^3^, corresponding to about 5 × 10^8^ unit cells. Fig. 1(*a*) ▶ shows a scanning electron microscope (SEM) micrograph of crystals that were similarly processed except that, for the sake of electron microscopy, were cross-linked according to the protocol given by Falkner *et al.* (2005 ▶).

Data collection was performed at the P11 beamline at the third-generation synchrotron source PETRA III (DESY, Hamburg). During our experiment the synchrotron operated in 60-bunch mode. The beam generated by the 122-pole undulator was monochromated to 0.01% bandwidth and focused using a Fresnel zone plate (focal length of 600 mm) to about 9 µm horizontally by 6 µm vertically. The X-ray energy was 9800 eV (1.27 Å wavelength) and the flux at the focus was 2 × 10^12^ photons s^−1^.

The suspension of crystals was pushed by a syringe pump (KDS LEGATO 200) through a 100 µm-inner-diameter fused silica fiber (Polymicro, USA) to a 100 µm-inner-diameter glass capillary (W. Müller, Germany) with 10 µm-thick walls that was mounted on a motorized stage with three orthogonal motions and placed horizontally in the X-ray interaction region. An in-line microscope was used to align the capillary to the beam and to observe the flow of crystals. A schematic of the experimental set-up is shown in Fig. 1(*b*) ▶. During measurements the sample was pushed at a constant rate, but the capillary was additionally scanned during this time to avoid accumulation of protein on the walls in the area illuminated by the X-ray beam. The scan covered a long rectangular area within the capillary that was at least 10 µm away from the top and bottom of the inner wall to avoid scattering from the edges of the capillary. The fast scan axis was parallel to the capillary axis. Scan speeds were slower than 0.1 mm s^−1^. Most measurements were performed at a liquid flow rate of 2.5 µl min^−1^, corresponding to an average flow velocity of 5 mm s^−1^ in the 100 µm-diameter capillary. For a perfectly laminar flow the velocity profile ranges from 3 mm s^−1^ at a distance of 10 µm from the inner wall to 10 mm s^−1^ at the center of the capillary. Thus, the transit times of crystals across the 9 µm-wide X-ray beam are estimated to vary between 1 and 3 ms, and are not significantly influenced by the scan speed. The calculated Reynolds number for the solution we used is 0.0115, well within the laminar flow regime. Even so, the suspended crystals were seen to affect the flow profile through collisions, tumbling and sedimentation, leading to a turbulent component to the flow that adds further uncertainty to the transit time and induces additional crystal rotation during the exposure. Indeed, rotation about an axis parallel to the beam direction (rolling) was observed occasionally in the form of the spreading of Bragg peaks into approximately constant radius arcs of about 5°. This is much larger than accountable by Brownian motion (estimated to be less than 0.01° in 3 ms) and is consistent with estimates of induced torque due to the inhomogeneous flow.

Diffraction patterns were collected using a Pilatus 6M detector placed at a distance of 300 mm from the interaction point, giving a resolution of 2.1 Å at the center edge of the detector. Detector frames were collected without the use of a mechanical shutter. The detector exposure time was 10 ms, but the frame rate was limited to 25 Hz by the readout time of the detector (a 75% dead-time). Given the beam size of 9 µm × 6 µm, a dose of 0.1 MGy would be accumulated in 3 ms, as estimated using the program *RADDOSE* (Paithankar *et al.*, 2009 ▶). The dose to a crystal was certainly no greater than 0.3 MGy, which would be an upper bound assuming that the crystal remains in the beam for the entire 10 ms detector exposure time.

## 3. Results   

### 3.1. Data collection and processing   

For our study we collected almost 1.5 × 10^6^ individual diffraction frames. This corresponded to an effective measurement time of about 17 h. With an average flow rate of 2.5 µl min^−1^, this resulted in a sample consumption of about 2.5 ml of crystal slurry or 250 mg of protein. These diffraction frames were then processed using *CrystFEL* (White *et al.*, 2012 ▶, 2013 ▶) version 0.5.3. The *CrystFEL* software suite was used to automatically index each pattern, providing the lattice vectors of the crystal oriented in the laboratory frame. In the framework of *CrystFEL*, this information was then used to predict the locations of Bragg reflections and obtain integrated and background-subtracted photon counts at these locations. Peak-finding thresholds and integration parameters were carefully tailored in order to maximize the number of properly indexed diffraction patterns. In more detail, the peak-finding algorithm used a simple gradient search after Zaefferer (2000 ▶) with a threshold of 25 photon counts. The intensity of each reflection was calculated by summing intensities within a radius of 2 pixels from the center of the predicted peak location, and subtracting the background signal estimated from an annulus with inner radius of 4 pixels and outer radius of 8 pixels.

A total of 40 233 patterns, or 2.7% of the total number of patterns acquired, were successfully indexed. However, taking into account only the patterns considered as the strongest (those with more than 15 Bragg peaks) and which were un­likely to be multiple-crystal hits (less than 200 Bragg peaks; see Fig. S1 of the supporting information), the percentage of indexed patterns was 24%.

Histograms of the lattice parameters are shown in Fig. S2. The mean values of these parameters (listed in Table 1 ▶) agree with the known values (*e.g.* Boutet *et al.*, 2012 ▶; Sauter *et al.*, 2001 ▶) within 0.5%. The reflection intensities were estimated using the Monte Carlo integration scheme (Kirian *et al.*, 2011 ▶) implemented in *CrystFEL*. For each indexed pattern this program predicts the locations of Bragg peaks, based on a model and the determined lattice parameters, and determines background-subtracted diffraction intensities, irrespective of whether a Bragg peak was detected. Peak locations were predicted assuming a detector located 300 mm away from the interaction point and a photon energy of 9800 eV, 0.01% bandwidth and beam divergence of 1 mrad.

**Figure 2:**
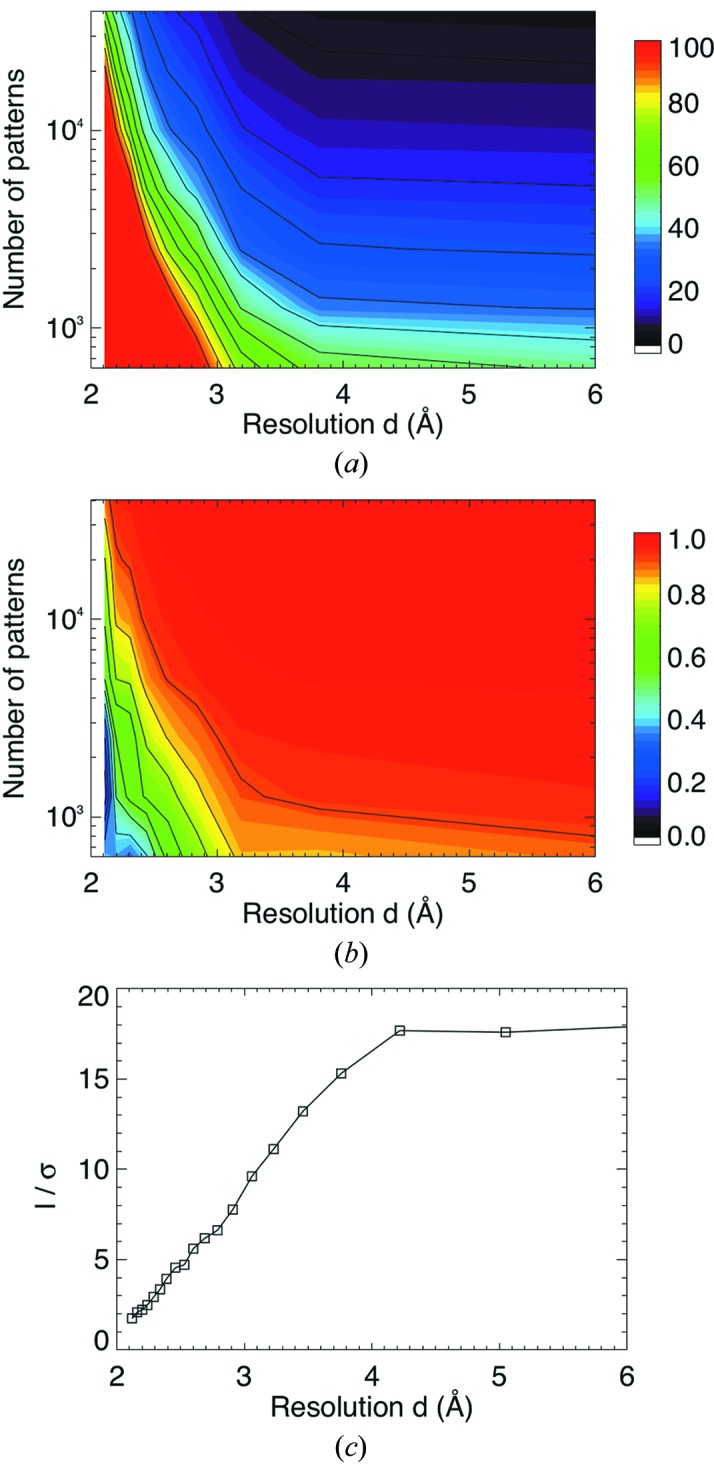
*R*_split_ (*a*) and 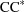 (*b*) plotted as a function of resolution and of number of indexed patterns. These are both metrics of internal consistency of the data. It can be seen that consistency is improved at a given resolution with an increase in the number of indexed patterns, and consistency is improved for a given number of indexed patterns by limiting the data to lower resolution. (*c*) Signal-to-noise ratio [*I*/σ(*I*)] of the merged data, averaged in resolution shells, plotted as a function of resolution. *I*/σ(*I*) of each reflection is defined as the mean counts divided by the standard error of those counts (White *et al.*, 2012 ▶).

The diffraction intensities of unique reflections in all indexed patterns were then averaged. As discussed above, this averages over many variations from crystal to crystal, including size, shape, exposure (transit) time and the partiality of the reflections. Although these factors lead to a broad distribution of measurements for each reflection, the ratio *I*/σ(*I*) can be estimated using the variance of the individual intensity measurements (White *et al.*, 2012 ▶). *I*/σ(*I*) values calculated in this way are shown in Fig. 2(*c*) ▶ as a function of resolution. This approach does not involve any scaling of the data and does not take into account factors such as possible lack of isomorphism of the sample, which can be due to twinning or to the presence of different crystal conformations. Sample (and beam) inhomogeneity affects data quality, but, thanks to the Monte Carlo integration of intensities, in the limit of a sufficiently large quantity of data, all stochastic variables are ‘integrated out’ and become constant factors affecting all intensities equally (White *et al.*, 2012 ▶).

### 3.2. Data consistency analysis   

The internal consistency of the merged data was judged on the basis of two resolution-dependent quantities that estimate the error of the Monte Carlo sum of intensities. The first, denoted *R*_split_, was computed as a crystallographic *R* factor, similar to *R*_merge_, that compares the intensities obtained from one half of the data (chosen at random) with those obtained from the other half (White *et al.*, 2012 ▶). The second metric, 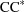, was determined from the Pearson correlation coefficient of the same two halves of the data, and then transformed to estimate the correlation of the full dataset to the (unknown) fully converged dataset (Karplus & Diederichs, 2012 ▶).

We examined the *R*_split_ and 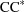 metrics as a function of resolution and number of merged diffraction patterns, plotted in Figs. 2(*a*) and 2(*b*) ▶, respectively. For the full dataset we find that the 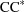 parameter drops from near-perfect correlation at low resolution to a value of 0.90 at 2.1 Å resolution and *R*_split_ ranges from 6% at low resolution to 53% at 2.1 Å, which we considered as the resolution limit. This limit shifts to lower resolution as the number of merged patterns is reduced. Occasionally we observed diffraction spots up to 2.1 Å resolution or higher, such as can be seen in the diffraction pattern of Fig. 1(*c*) ▶. In this pattern the highest resolution Bragg peak used for merging contained 33 ± 7 photons, where the error is due to the noise from the subtracted background. The prevalence of such patterns can be estimated from a plot of the total integrated Bragg counts in a pattern as a function of the highest resolution observed in that pattern, showing that, as expected, the highest resolution peaks tend to coincide with the patterns that have more Bragg peak photons in total (see Fig. S3). This observation, and the plots of *R*_split_ and 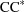, suggest that resolution could be improved by the inclusion of yet more data, implying that the limit we observe is not an inherent property of the crystals, but is a consequence of the achieved accumulated signal level relative to the noise in the background.

### 3.3. Electron-density determination   

The merged intensities obtained by *CrystFEL* from the 40 233 indexed patterns were converted to MTZ format for further processing. As listed in Table 1 ▶, the Wilson *B* factor of our data is 44.1 Å^2^ (as determined by *phenix.xtriage*), which is higher than typically obtained for single-crystal room-temperature data. This high *B* factor might be attributable to our choice of merging intensities from all indexed patterns at predicted spot locations out to the edges of the detector (that is, at scattering angles that are often beyond the highest resolution observed spots in a pattern). This choice may improve the accuracy of structure factors, by averaging weak signals, but certainly lowers estimated integrated intensities at high resolution compared with averaging only counts above a given threshold.

**Figure 3:**
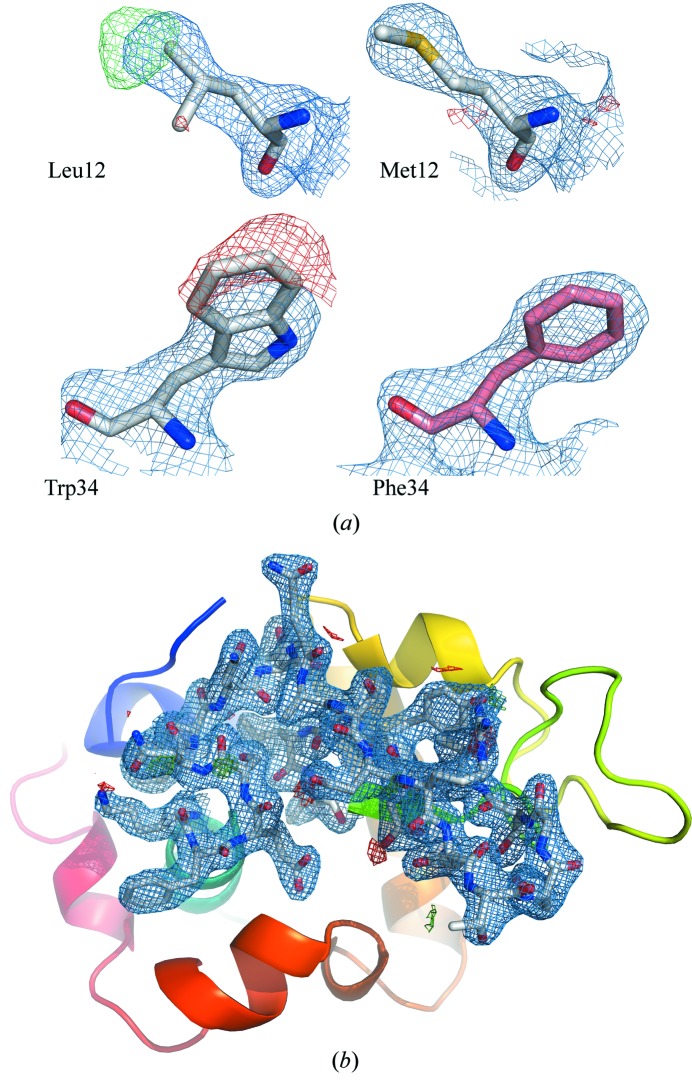
(*a*) Detail of the electron-density map showing 2*m* *F*_o_ − *D* *F*_c_ (1.0σ) overlaid with *m* *F*_o_ − *D* *F*_c_ (2.5σ). The left-hand panels show close-ups centered on residues Leu12 and Trp34 of the human protein. The right-hand part shows that a better fit of electron density is reached when these residues are mutated into those present in chicken egg-white lysozyme, namely Met12 and Phe34. (*b*) Electron-density map (2*m* *F*_o_ − *D* *F*_c_ at 1σ overlaid with *m* *F*_o_ − *D* *F*_c_ at 2.5σ) of lysozyme at 2.1 Å resolution calculated from 40 233 single-crystal indexed diffraction patterns. The electron-density map covers the residues between 33 and 55.

The data were phased by molecular replacement (MR) with *PHASER* (McCoy *et al.*, 2007 ▶) using a search model generated from human lysozyme (PDB entry 2zil) using *phenix.pdbtools* (Adams *et al.*, 2010 ▶) (LLG = 629 and TFZ = 17.6). The differences in the sequence between the human lysozyme used as a model and the chicken lysozyme gave rise to clearly visible differences in the electron-density map (2*m* *F*_o_ − *D* *F*_c_ overlaid with *m* *F*_o_ − *D* *F*_c_, shown in Fig. 3*a* ▶). Automated model (re-)building with prime-and-switch phasing (Terwilliger, 2004 ▶) was then carried out using *phenix.autobuild* (Terwilliger *et al.*, 2008 ▶) resulting in a completely built lysozyme model (*R*_work_ = 18.5%, *R*_free_ = 24.0%). This was further subjected to iterative cycles of restrained refinement with simulated annealing using *phenix.refine* (Afonine *et al.*, 2012 ▶) and model building with *Coot* (Emsley *et al.*, 2010 ▶) followed by a final refinement carried out with *PDB_REDO* (Joosten *et al.*, 2011 ▶) using *REFMAC* (Murshudov *et al.*, 2011 ▶). It resulted in a refined model (*R*_work_ = 17.6%, *R*_free_ = 23.0%) at 2.1 Å resolution. The refinement and structural validation statistics are given in Table 1 ▶ and the electron density is shown in Fig. 3(*b*) ▶. An iterative build composite map was generated using *phenix.autobuild* (see Fig. S4) to assess the quality of our collected data.

We performed further analysis to determine the dependence of refinement on the number of merged indexed patterns, by generating sub-datasets of randomly chosen patterns. To avoid bias in these comparisons, we processed all sub-datasets in the same way without any manual refinement of the electron densities. The values of *R*_work_ and *R*_free_ obtained after restrained refinement for the different numbers of patterns are plotted in Fig. S5. These plots suggest that at least 5000 patterns, that under the current experimental conditions could be measured in less than 2 h, are required to obtain satisfactory *R*_work_ and *R*_free_ values.

## 4. Discussion and conclusions   

The work presented here is a proof-of-principle demonstration that room-temperature serial crystallography measurements can be performed at a high-brightness synchrotron and can be used to solve the structure of a protein from tens of thousands of indexed single-crystal diffraction patterns. Our work is a step towards adapting the methodology of serial crystallography, which has been developed recently for free-electron laser sources, for room-temperature synchrotron data collection. The methods described here can equally be applied to any serial sample delivery technique including free-flowing liquid jets without a capillary, membrane proteins embedded in lipidic cubic phase medium extruded into the X-ray focus, and microcrystals deposited on fixed targets scanned through the X-ray beam.

Improving the sample delivery system and the beamline set-up will allow this approach to be used to collect data from smaller crystals with more efficient sample consumption and shorter data collection time as well as shorter crystal exposure times. Improvements can be made to reduce background in measurements by better matching the detector exposure time to the crystal transit time or by using polycarbonate capillaries with smaller inner diameters, for example (see also §1 and Fig. S6 in the supporting information). Other delivery systems, such as an extruded jet of viscous medium (Liu *et al.*, 2013 ▶; Weierstall *et al.*, 2014 ▶) or X-ray compatible microfluidic chips (Brennich *et al.*, 2011 ▶; Weinhausen & Köster, 2013 ▶; Nielsen *et al.*, 2012 ▶; Emamzadah *et al.*, 2009 ▶; Heymann *et al.*, 2014 ▶; Pinker *et al.*, 2013 ▶; Guha *et al.*, 2012 ▶), could also produce much lower background than in our experiments. We note that since measurements are conducted in air (as opposed to a vacuum for X-ray FEL serial crystallography experiments) the sample can be easily collected and possibly recycled.

There are several advantages of the serial approach as compared with traditional methods. Assuming errors are dominated by photon counting and background, the estimation of a structure factor improves the more measurements that can be averaged. The strategy of flowing crystals rather than mounting them on a goniometer could lead to structure determination in a fully automated way, using apparatus to dispense samples similar to that used for continuous X-ray solution scattering (Franke *et al.*, 2012 ▶). The method is scalable to much faster data collection that will become possible with upcoming upgrades of third-generation synchrotron facilities. For example, plans at the European Synchrotron Radiation Facility (ESRF) are to increase the source brightness by a factor of 1000. Such improvements will be of little help for conventional crystallography, but would allow serial crystallography to be conducted at higher flow rate with microsecond crystal transit times and a corresponding shortening of the entire measurement time. Indeed, the P11 beamline where we undertook this work will be soon upgraded by implementing a pink-beam high-flux operating mode that will deliver up to 10^15^ photons s^−1^. In this set-up the tolerable exposure time for a crystal will be of the order of microseconds, requiring a flow speed of about 1 m s^−1^ which could be obtained with a gas dynamic virtual nozzle (DePonte *et al.*, 2008 ▶), for example. In that case diffraction data will be acquired at over 100 counts pixel^−1^ s^−1^, so high-frame-rate integrating detectors will be needed. A possible option is the adaptive gain integrating pixel detector (AGIPD) under development for use at the European XFEL (Henrich *et al.*, 2011 ▶; Becker *et al.*, 2013 ▶). Moreover, the higher bandwidth of the pink beam (1.5%) compared with a monochromatic beam will allow the measurement of more reflections in each pattern and to reduce the effect of reflection partiality on the data (Dejoie *et al.*, 2013 ▶; White *et al.*, 2013 ▶), thus leading to a smaller number of patterns required.

Our method avoids cryogenic cooling, which is known to lead to structural artefacts or over-interpretation of diffraction data (Fraser *et al.*, 2011 ▶). Although cryocooling usually does not modify the overall structure of a protein, the dynamic properties of the protein may be changed (Rasmussen *et al.*, 1992 ▶) and structures collected at 100 K can deviate significantly from the biologically relevant active form. Moreover, room-temperature measurements do not require the use of cryoprotectants, thus allowing the crystals to be used in their native buffer without further manipulation.

At room temperature, there is evidence that faster exposures give rise to a larger tolerable dose (Blake *et al.*, 1962 ▶; Southworth-Davies *et al.*, 2007 ▶; Owen *et al.*, 2012 ▶; Warkentin *et al.*, 2011 ▶, 2013 ▶). Owen *et al.* (2012 ▶) studied the effect of dose rate at room temperature on crystals of a soluble protein, a virus and a membrane protein. In all three cases they observed an increase in the life dose of the crystals with an increase of beam intensity (photons per area per time) and corresponding decrease in exposure time. They attribute this effect to the ability to collect diffraction data before hydroxyl radicals can propagate through the crystal, disordering the structure. Warkentin *et al.* (2011 ▶) showed that global damage happens on a timescale of the order of seconds, and that damage increases with time after the X-rays have been turned off, an effect called ‘dark progression’. Our method is ideally suited to perform a series of very short exposures, from microseconds up to a few milliseconds, to take advantage of higher dose rates. It is also worth noting that radiation damage could be significantly lowered by reducing crystal sizes down to micrometre size, owing to an escape of the secondary electrons from the crystal (Nave & Hill, 2005 ▶; Holton & Frankel, 2010 ▶; Sanishvili *et al.*, 2011 ▶).

Finally, the serial crystallography approach is naturally well suited for time-resolved experiments on millisecond timescales (or faster when shorter exposures are possible), including measurements of structural changes in irreversible reactions (Aquila *et al.*, 2012 ▶). The study of irreversible reactions requires a fresh crystal for each time point and orientation, which can make the standard experiments impractical. Additionally, smaller crystals have shorter times for a substrate molecule to fully diffuse through the crystal, giving faster time resolution in a mixing experiment (Schmidt, 2013 ▶).

## Supplementary Material

PDB reference: lysozyme by serial crystallography, 4o34

Supporting information figures S1 to S6. DOI: 10.1107/S2052252514010070/it5001sup1.pdf

## Figures and Tables

**Table 1:** Data statistics including shells up to 2.1 Å resolution

Data collection	
Light source, beamline	PETRA III, P11
Maximum dose (MGy)	0.3
Space group	*P*4_3_2_1_2
Cell dimensions *a*, *b*, *c* (Å)	79.5 ± 0.3, 79.4 ± 0.3, 38.4 ± 0.2
*V*_M_ (Å^3^ Da^−1^)/solvent content (%)	2.07/40.6
Resolution range (Å)	39.65–2.09
Wilson *B* factor (Å^2^)	44.1
Completeness (%)	93.4 (82.0)
*R*_split_	7.65 (53.98)
*I*/σ(*I*)	8.1 (1.9)
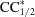	0.9986 (0.9007)
Redundancy	1755 (1281)

Refinement	
PDB ID	4o34
Resolution range (Å)	39.65–2.09
No. reflections used in refinement	6411
No. reflections used for *R*_free_	709
*R*_work_/*R*_free_	0.18/0.23 (0.28/0.30)
No. atoms	
Protein	1000
Ions	3
Water	12
*B* factors (Å^2^)	
Protein	51.7
Ions	59.1
Water	45.1
R.m.s. deviations	
Bond lengths (Å)	0.007
Bond angles (°)	1.08
Ramachandran plot (%)	
Most favored	97.6
Allowed	2.4
Disallowed	0.0
