# Supplementary material for: Room-temperature macromolecular serial crystallography using synchrotron radiation
Source: IUCrJ. 2014 May 30;1(Pt 4):204–12. doi: 10.1107/S2052252514010070 (PMC4107920; doi:10.1107/S2052252514010070)

**IUCrJ**

**Volume 1 (2014)**

**Supporting information for article:**

# Room-temperature macromolecular serial crystallography using synchrotron radiation

**Francesco Stellato, Dominik Oberthür, Mengning Liang, Richard Bean, Cornelius Gati, Oleksandr Yefanov, Anton Barty, Anja Burkhardt, Pontus Fischer, Lorenzo Galli, Richard A. Kirian, Jan Meyer, Saravanan Panneerselvam, Chun Hong Yoon, Fedor Chervinskii, Emily Speller, Thomas A. White, Christian Betzel, Alke Meents and Henry N. Chapman**

**Figure S1** Distribution of the number of detected peaks in the 40233 indexed patterns. The average number of Bragg peaks detected in the indexed patterns is 42.

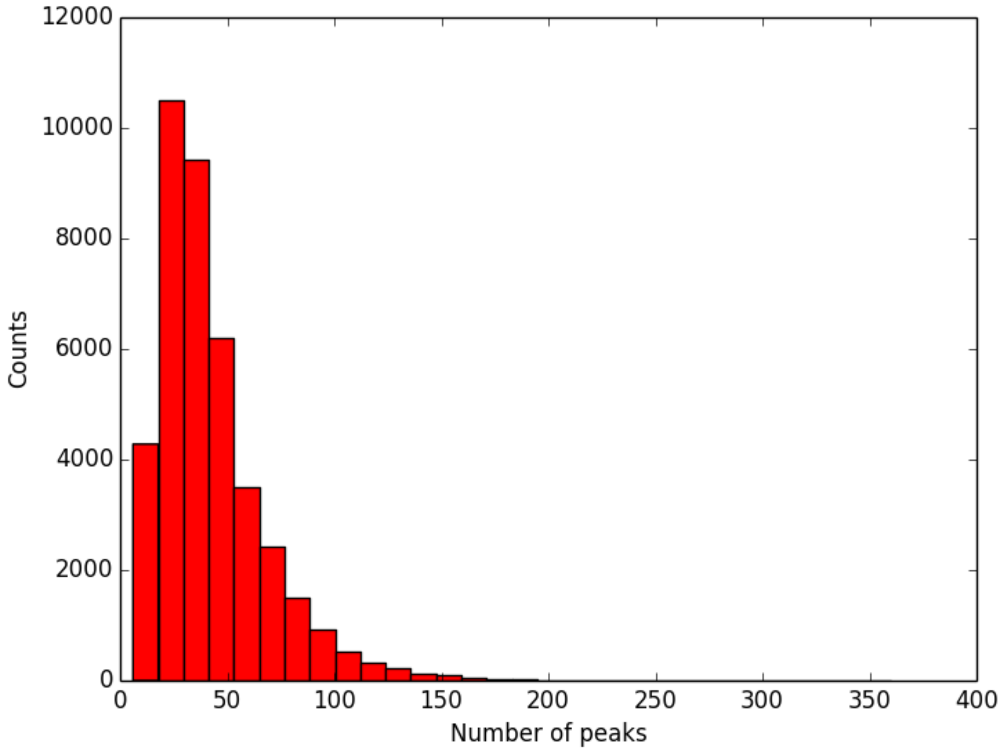

**Figure S2** Distributions of unit cell parameters determined from the 40233 indexed patterns. The patterns were indexed providing the known unit cell parameters of lysozyme to the indexing algorithms and giving a tolerance of 5% on the axes and 1.5 degrees on the angles.

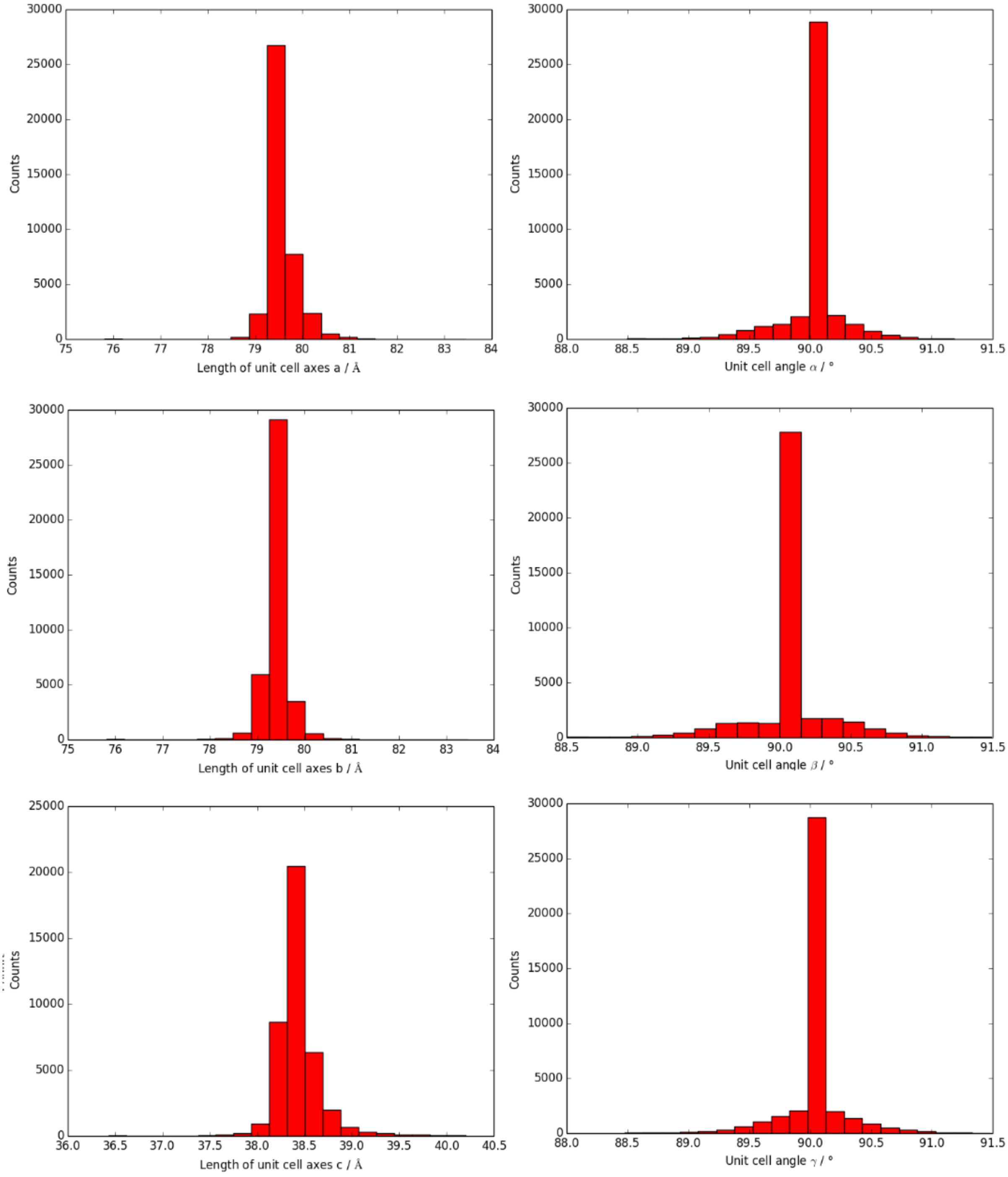

**Figure S3** Scatter plot of the total integrated Bragg counts in all Bragg peaks in an indexed pattern as a function of the highest resolution observed in that pattern. This plot indicates that, as expected, the highest resolution peaks tend to coincide with the brightest patterns.

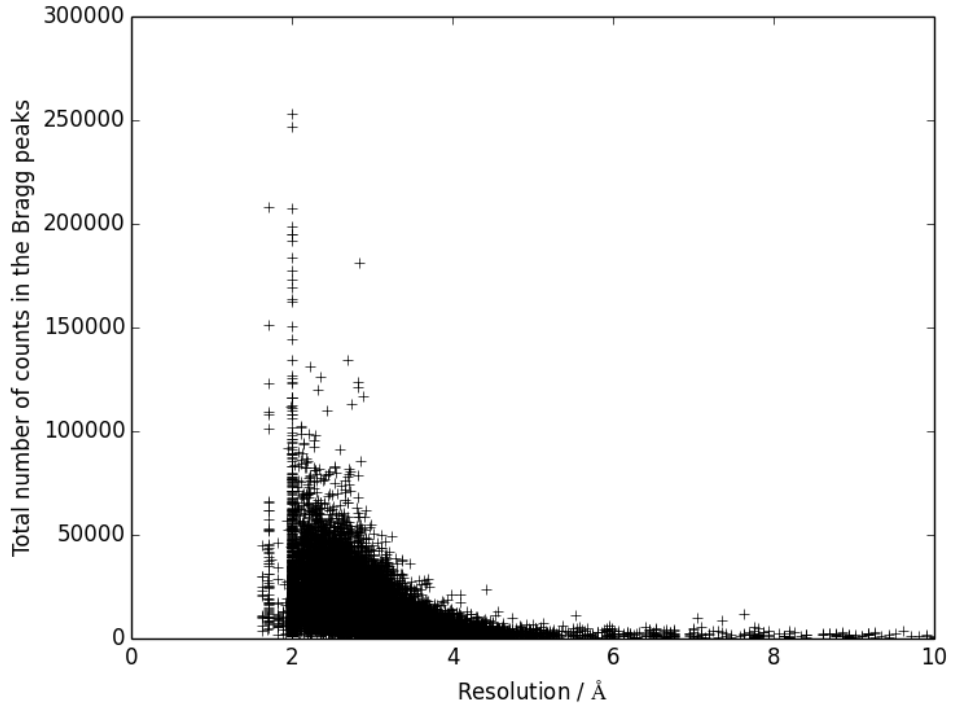


**Figure S4** To assess the quality of the collected data an iterative build composite omit map was created with phenix.autobuild. Here we show the resulting map for residues 33 to 55 of chicken egg white lysozyme (contoured at 1σ).

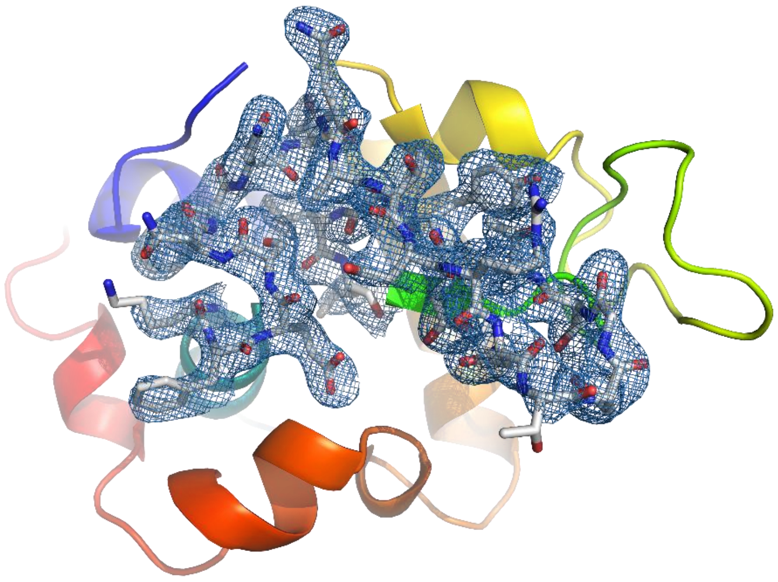

**Figure S5** Structural refinement as a function of number of indexed patterns and resolution.

(a) $R_{work}$ and $R_{free}$ plotted as a function of the number of indexed patterns used for the merging of intensities. The values are calculated including the shells up to 2.1 Å resolution. (b) $R_{work}$ and $R_{free}$ obtained merging the intensities of 40233 patterns, plotted as a function of the maximum resolution shell used for the refinement.

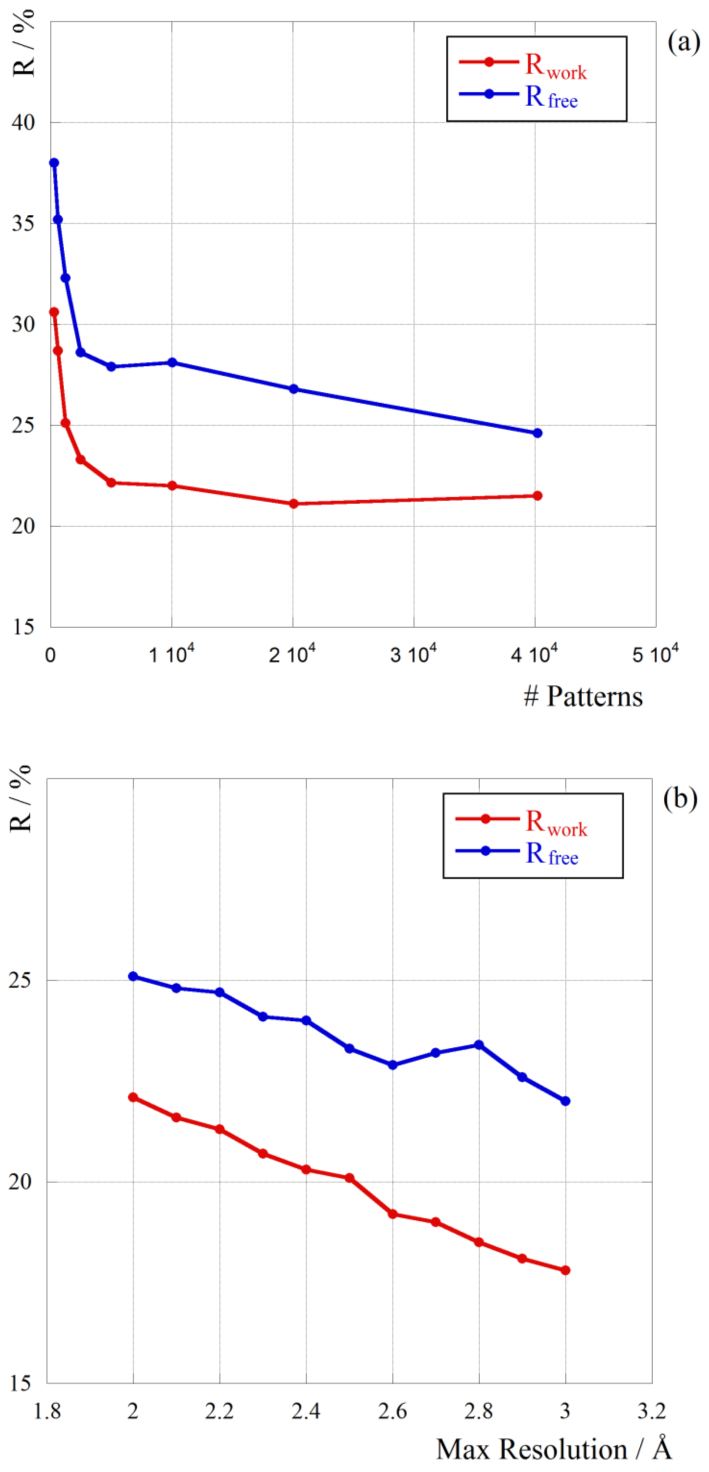

### S1. Background analysis

For this proof-of-principle measurement we used thin-walled glass capillaries that are commonly used for SAXS measurements. Figure S6 shows a pattern collected from an empty capillary and a total air-path of 300 mm. The glass and air contribution is strongest between resolutions of 4 and 3 Å, at a level of about 10 photon counts / pixel / 10 ms. This compares with Bragg counts of 30 photon counts / pixel / 10 ms for crystals that we used for this experiment, allowing adequate measurement of the peaks in this case. For smaller crystals further developments are being considered, including the use of thinner wall capillaries or of capillaries of other materials.

**Figure S6**  A diffraction pattern showing the background due to the air path and to the glass capillary.

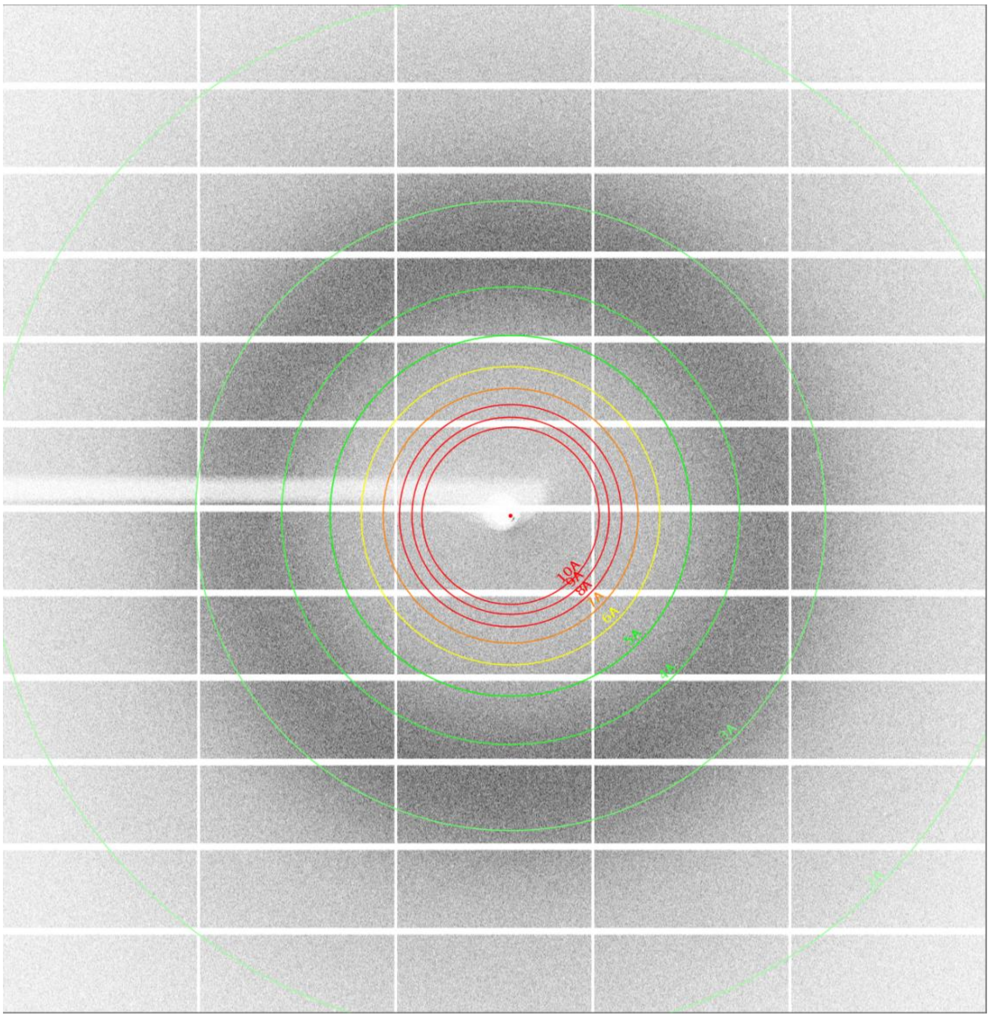

Supplement: Supplementary file 1 [file m-01-00204-sup1.pdf]
